# Exercise and global well-being in community-dwelling adults with fibromyalgia: a systematic review with meta-analysis

**DOI:** 10.1186/1471-2458-10-198

**Published:** 2010-04-20

**Authors:** George A Kelley, Kristi S Kelley, Jennifer M Hootman, Dina L Jones

**Affiliations:** 1 Department of Community Medicine, West Virginia University, Morgantown, West Virginia, USA; 2 Centers for Disease Control & Prevention, Atlanta, Georgia, USA; 3 Department of Orthopaedics and Division of Physical Therapy, West Virginia University, Morgantown, West Virginia, USA

## Abstract

**Background:**

Exercise has been recommended for improving global-well being in adults with fibromyalgia. However, no meta-analysis has determined the effects of exercise on global well-being using a single instrument and when analyzed separately according to intention-to-treat and per-protocol analyses. The purpose of this study was to fill that gap.

**Methods:**

Studies were derived from six electronic sources, cross-referencing from retrieved studies and expert review. Dual selection of randomized controlled exercise training studies published between January 1, 1980 and January 1, 2008 and in which global well-being was assessed using the Fibromyalgia Impact Questionnaire (FIQ) were included. Dual abstraction of data for study, subject and exercise program characteristics as well as assessment of changes in global well-being using the total score from the FIQ was conducted. Risk of bias was assessed using the Cochrane bias assessment tool. Random-effects models and Hedge's standardized effect size (*g*) were used to pool results according to per-protocol and intention-to-treat analyses.

**Results:**

Of 1,025 studies screened, 7 representing 5 per-protocol and 5 intention-to-treat outcomes in 473 (280 exercise, 193 control) primarily female (99%) participants 18-73 years of age were included. Small, statistically significant improvements in global well-being were observed for per-protocol (*g *and 95% confidence interval, -0.39, -0.69 to -0.08) and intention-to-treat (-0.34, -0.53 to -0.14) analyses. No statistically significant within-group heterogeneity was found (per-protocol, Q_w _= 6.04, *p *= 0.20, *I*^2 ^= 33.8%; intention-to-treat, Q_w _= 3.19, *p *= 0.53, *I*^2 ^= 0%) and no between-group differences for per-protocol and intention-to-treat outcomes were observed (Q_b _= 0.07, *p *= 0.80). Changes were equivalent to improvements of 8.2% for per-protocol analyses and 7.3% for intention-to-treat analyses.

**Conclusions:**

The results of this study suggest that exercise improves global well-being in community-dwelling women with fibromyalgia. However, additional research on this topic is needed, including research in men as well as optimal exercise programs for improving global well-being in adults.

## Background

Fibromyalgia is a chronic rheumatic condition characterized by widespread pain, fatigue, and multiple tender points [1]. A recent study by the National Arthritis Data Workgroup estimated the prevalence of primary fibromyalgia to be approximately 5 million among US adults 18 years of age and older in 2005 [2]. Approximately 87% of those diagnosed with fibromyalgia in the US are women (population prevalence = 3.4% females versus 0.5% males), with diagnosis most often occurring during middle age [2]. Adults with fibromyalgia have 2-3 times higher healthcare costs [3,4] and report poorer well-being (16.5% to 52% lower scores on the SF-36) compared to healthy persons [5,6].

Exercise is a non-pharmacologic intervention that has been recommended for community-dwelling adults with fibromyalgia [1]. One of the most commonly measured outcomes when assessing the effects of exercise in those with fibromyalgia is global well-being [7]. Recent randomized controlled trials examining the effects of exercise on global well-being among those with fibromyalgia have yielded conflicting findings [8-14]. For example, using the total score from the Fibromyalgia Impact Questionnaire (FIQ), the most commonly used instrument for assessing global well-being in fibromyalgia participants [7], four studies reported a statistically significant improvement in global well-being [8,9,12,14], while another three reported no statistically significant improvement [10,11,13].

A previous systematic review [7] examined the effects of exercise on global well-being using the total score from the FIQ, study participant-rated change in fibromyalgia symptoms and observer-rated changes in fibromyalgia symptoms. The authors reported statistically significant improvements as a result of aerobic exercise (standardized mean difference, 0.49, 95% confidence interval, 0.23 to 0.75) and strength training (standardized mean difference, 1.43, 95% confidence interval, 0.76 to 2.10). These were equivalent to relative improvements in global well-being of 12% for aerobic exercise from 4 studies [9,10,13,15] and 122% for strength training from only 2 studies [16,17]. Generally, recommendations regarding the clinical importance of relative improvements have ranged from 15% [18] to 30% [19].

Since the time of the last search for the previous review (July, 2005), additional randomized controlled trials on this topic have been published [11,12,14], with only one of the three studies reporting a statistically significant improvement in global well-being based on the total score from the FIQ [12]. In addition, the authors of the previous systematic review included multiple measures of global well-being [7], an approach that may be problematic given that recent research has suggested that the pooling of global well-being instruments could result in biased meta-analyses [20]. More specifically, the use of different instruments to assess global well-being might attenuate any estimated effects or add spurious between-study variance because of between-measure "noise." Furthermore, the authors preferentially included results based on intention-to-treat analyses if they were available despite the fact that the opportunity existed to examine intention-to-treat and per-protocol results separately. This may be problematic given that recent meta-epidemiological research found that excluding participants from randomized controlled trials in systematic reviews often results in biased estimates of treatment effects [21]. Based on this finding, the authors recommended that systematic reviews routinely assess the influence of the exclusion of participants on estimated treatment effects [21].

Thus, given that (1) additional randomized controlled trials on the effects of exercise on global well-being have been published since the last systematic review [7], (2) the potential problems in pooling results from different measures of global well-being [20], and (3) the fact that no one to date has examined per-protocol and intention-to-treat results separately, the purpose of this study was to use the meta-analytic approach to examine the effects of exercise on global well-being as assessed by the FIQ in community-dwelling adults with fibromyalgia.

## Methods

### Data Sources and Searches

Studies for the current meta-analysis were retrieved from a large in-house and broad exercise and rheumatic disease database that includes 1025 citations. This initial database was developed by searching six electronic sources (PubMed, EmBase, Cochrane Central Register of Controlled Clinical Trials, CINAHL, SPORTDiscus and Dissertation Abstracts Online), cross-referencing from retrieved studies, including review articles, and expert review (Dr. Miriam Nelson, Tufts University, personal communication, June 13, 2008). All computer searches were conducted by the second author with the assistance of the first author. Search queries for the six electronic sources from which studies were derived for this database are shown in Additional File 1. From the 1025 citations in the database, a search for studies dealing with the effects of exercise on global well-being using the FIQ in participants with fibromyalgia was conducted using the single keyword "fibromyalgia" while searching across all indexed fields within the database.

### Study Selection

The inclusion criteria for this study were: (1) randomized controlled trials with the unit of assignment at the participant level, (2) an exercise-only intervention group (aerobic, strength training, or both), (3) community-accessible exercise interventions, defined as those interventions that could be performed and made available to non-institutionalized persons in a community setting, (4) exercise intervention of at least four weeks, (5) a comparative control group (usual care or attention control), (6) community-dwelling adults aged 18 years and older with fibromyalgia, (7) published and unpublished studies (master's degrees and dissertations), (8) studies published in any language between January 1, 1980 and January 1, 2008, and (9) data available on global well-being as assessed by the total score from the FIQ [22]. The FIQ, described in detail elsewhere [22], is a self-administered instrument aimed at assessing global well-being in participants with fibromyalgia. It takes approximately 5 minutes to complete [22]. The validity and reliability of the FIQ has been previously established, including its ability to detect therapeutic change [22]. Studies were limited to those in which aerobic and/or strength training were the only interventions because they are the two most common nonpharmacologic interventions recommended for maintaining overall fitness [23-25] and are most likely to be performed in the community. In addition, to answer the research question for this study, the difference between the experimental and control group had to be the exercise intervention in the experimental group. The search for studies started in 1980 based on the recommendations from rheumatic disease experts associated with this project as well as an external consultant (Dr. Miriam Nelson, Tufts University, personal communication, June 13, 2008). In addition, this was the approximate time in which the FIQ was first developed [22]. All trials other than randomized controlled trials were excluded because nonrandomized trials cannot fully control for confounders that are not known or measured and have been shown to overestimate estimates of treatment effects [26,27]. Review articles were excluded because they did not contain complete data for each study reviewed. Rather, cross-referencing from review articles was performed to identify studies that might be included. Follow-up studies in which the initial intervention ended but participants continued to be tracked post-intervention were excluded because the focus of this project was on the exercise intervention versus exercise behavior post-intervention. Since the focus of the current meta-analysis was on participants with fibromyalgia, studies in participants without fibromyalgia were excluded. Studies less than 4 weeks were excluded based on the expectation that exercise-induced changes in global well-being, if any, might be reasonably expected to occur by this time. To avoid multiple publication bias, studies that included the same subjects as another study were excluded. Trials that did not collect and report data using the FIQ were excluded based on previous research suggesting that the pooling of global well-being instruments could result in biased meta-analyses [20]. Abstracts were excluded because of the minimal information provided and the difficulty in retrieving this information. Acute studies, defined as those studies in which global well-being was assessed immediately after a single-exercise session, were excluded given the research teams interest in the chronic effects of exercise on global well-being. Studies in children and/or adolescents were excluded because of the many maturational changes that occur during this time. Finally, rehabilitation studies were excluded because of the investigative team's interest in exercise programs that could be replicated in the community, a public health approach that might have the greatest reach in terms of participants with fibromyalgia. The selection of studies was conducted by the first two authors. Using Cohen's kappa statistic [28], the overall agreement rate (yes/no based on whether to include or exclude) prior to adjudication was 0.89.

### Data Abstraction

Prior to the abstraction of data, a codebook was developed that included the following major categories: (1) study characteristics, (2) subject characteristics, (3) exercise program characteristics and (4) outcomes (e.g., changes in global well-being as assessed by the total score from the FIQ). All studies were coded by the first two authors, independent of each other. The authors then reviewed every item for accuracy and precision. Disagreements were resolved by consensus. Using Cohen's kappa statistic [28], the overall agreement rate prior to correcting discrepant items was 0.96, considered to be almost perfect [29].

### Risk of Bias Assessment

Because of the lack of empirical evidence [27,30], including validity [31], to support the use of quality scales, the risk of bias assessment tool recently recommended by the Cochrane Collaboration was used to assess bias across six domains: (1) sequence generation, (2) allocation concealment, (3) blinding to group assignment, (4) incomplete outcome data, (5) selective outcome reporting, and (6) other potential bias [32]. Each domain was classified as having either a high, low, or unclear risk of bias [32]. The decision rule for blinding was that participants, research personnel, and outcome assessors were blinded to the primary outcome of interest, that is, global well-being as assessed by the FIQ. Blinding of all three groups was considered important given the subjective nature of measures of global well-being. Selective reporting was also limited to global well-being as assessed by the FIQ and was based on the outcome being reported in the methods and data in the results [33]. Other potential forms of bias were limited to between-group differences in baseline global well-being as assessed by the FIQ. All assessments were conducted by the first two authors, independent of each other. Both authors then met and reviewed every item for agreement. Disagreements were resolved by consensus. Using Cohen's kappa statistic [28], overall inter-rater agreement prior to correcting discrepant items was 0.10, considered to be slight [29]. On an itemized basis and using the general categories suggested by Landis [29], inter-rater agreement was 1.0 (perfect) for between-group baseline differences in the FIQ, 0.71 (substantial) for sequence generation, 0.14 (slight) for allocation concealment and incomplete outcome reporting, and 0 (poor) for blinding and selective outcome reporting. The range of inter-rater agreement for each domain was similar but wider than recent methodological research using this assessment tool (0.13 to 0.74) [33]. The wide range of inter-rater reliability scores is most likely due to the degree of subjective decision-making that is allowed with this tool, and for the current study, the fact that this was the first time that the research team used this instrument.

### Data Synthesis and Analysis

#### Calculation of study-level effect-size estimates for the FIQ

The primary outcome in this study was the total score from the FIQ [22]. Given the different versions and methods of reporting, the standardized effect size (*g*) was used for all FIQ outcomes [34]. For the one study that included two exercise groups[13], *g *was pooled in order to maintain independence. Since all studies were parallel trials, the *g *for each outcome from each study was calculated as the difference in change scores between the exercise and control groups divided by the pooled standard deviations of these change scores [34]. This calculation included an adjustment for small within-group sample sizes [34]. Since change score standard deviations were not reported for any of the included studies, these were estimated for six studies [9-14] using pre- and post-intervention means and standard deviations in the exercise and control groups [35]. Standard deviations were calculated for another study using the 95% confidence intervals that were reported [8]. The variance of each *g *from each study was then estimated using previously developed procedures [34]. Since a lower total FIQ score represents higher global well-being, a negative *g *indicates that exercise improved global well-being in participants.

#### Pooled estimates for FIQ

A random effects model was used to pool FIQ outcomes from each study and were reported according to whether the data were analyzed using a per-protocol or intention-to-treat approach. If the two-tailed 95% confidence intervals generated from the models did not cross zero, results were considered to be statistically significant. In terms of magnitude, values for *g *of 0.20, 0.50, and 0.80 have been suggested to represent small, medium, and large effect sizes [36]. Heterogeneity of FIQ outcomes between studies was examined using the *Q *statistic and a commonly used alpha value for statistical significance of 0.10 [34]. In addition, the consistency of between-study findings for FIQ outcomes were analyzed using *I*^2^[37]. Generally, *I*^2 ^values of 25%, 50%, and 75% may be considered to represent small, medium, and large amounts of inconsistency. For this study, the decision rule for heterogeneity was a *Q*_w _value ≤ 0.10 and/or an *I*^2 ^value greater than 50%. In addition, mixed-effects models were used to test for between-group differences (Q_b_) in FIQ outcomes according to per-protocol and intention-to-treat analysis. Since there was no statistically significant heterogeneity for per-protocol or intention-to-treat results and the number of included studies was small, no analyses for potential covariates were conducted. An alpha value of ≤ 0.05 was considered to be statistically significant for the between-group comparison.

In order to enhance interpretability, the common language effect size (CLES) was calculated for all FIQ outcomes [38]. Using the CLES, a *g *of 0.50, for example, means that 64% of the subjects in an experimental group will score higher than subjects in a control group if chosen at random. In addition, *g *was converted to an odds ratio (OR) to further enhance interpretation.

#### Publication bias

Publication bias was examined using the nonparametric trim and fill linear estimator *L *approach of Duvall and Tweedie [39].

#### Sensitivity analysis

In order to examine the influence of each study on the overall results, each study was deleted from the model once and the pooled analyses conducted with that one study deleted from the model. Because of the small number of studies as well as the lack of between-study heterogeneity, bias assessment results were not incorporated into the statistical analysis of data.

#### Cumulative meta-analysis

In order to examine changes in findings over time, cumulative meta-analysis, ranked by year, was performed [40]. Cumulative meta-analysis is an approach in which study results are added one at a time in a specified order and summarized as each new study is added [40].

#### Software utilization for statistical analysis

Descriptive statistics were generated using SPSS (version 16.0) [41]. All meta-analytic analyses were conducted using Comprehensive Meta-Analysis (version 2.2) [42].

## Results

### Study Characteristics

**Figure 1:**
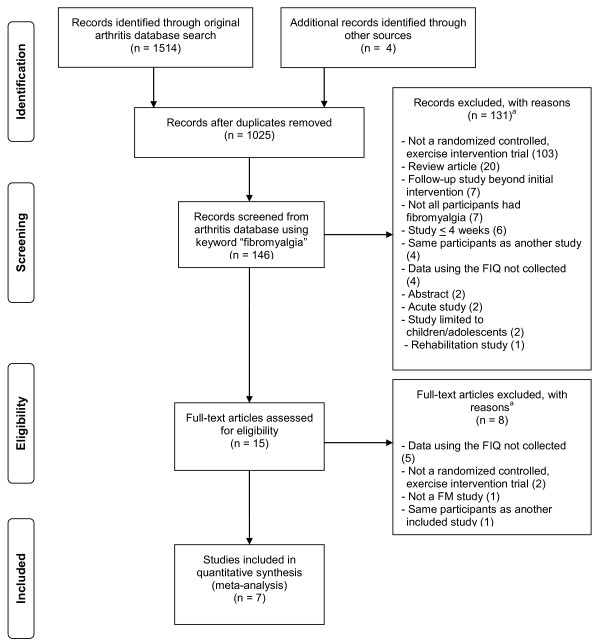
**Flow diagram for the selection of studies**. ^a^The number of reasons for exclusion exceeds the number of studies excluded because some studies were excluded for multiple reasons.

Of the 1,025 studies screened, 7 representing 10 FIQ outcomes (5 per-protocol and 5 intention-to-treat scores) were included (Figure 1) [8-14]. All of the studies were published in English-language journals between 2001 and 2007 [8-14]. Four studies were conducted in Canada [8-10,13], two in Spain [12,14] and one in the United States [11]. Another four studies reported using both per-protocol and intention-to-treat approaches in the analysis of their data [9-11,13], two were limited to per-protocol [12,14] and one to intention-to-treat [8].

Results for risk of bias are shown in Table 1. Within each domain and across all studies, a low risk of bias for selective reporting of data using the FIQ as well as between-group differences in baseline FIQ was observed. Alternatively, all studies were considered to be at a high risk of bias for blinding of participants, personnel and outcome assessors to group assignment. Adequate sequence generation was unclear in more than half of the studies while adequate allocation concealment was unclear in almost three fourths. Three fourths of studies were also considered to be at a low risk of bias for adequately reporting incomplete outcome data. Within each individual study and across all domains, low, high, and unclear risks of bias ranged, respectively, from 33% to 83%, 17% to 33%, and 0% to 50%.

**Table 1:** Risk of bias assessment.

Reference			Sequence Generation	Allocation Concealment	Blinding	Incomplete Outcome Data	Selective Reporting	Other Bias	Low Risk	High Risk	Unclear
Da Costa et al. (2005)[8]			Low	Low	High	Low	Low	Low	83%	17%	0%
Gowans et al.(2001)[9]			Unclear	Unclear	High	Unclear	Low	Low	33%	17%	50%
King et al. 2002)[10]			Low	High	High	Low	Low	Low	67%	33%	0%
Kingsley et al. (2005)[11]			Low	Unclear	High	Low	Low	Low	67%	17%	17%
Munguia-Izquierdo & Legaz-Arrese (2007)[12]			Unclear	Unclear	High	Unclear	Low	Low	33%	17%	50%
Schachter et al. (2003)[13]			Unclear	Unclear	High	Low	Low	Low	50%	17%	33%
Tomas-Carus et al. (2007) [14]			Unclear	Unclear	High	Low	Low	Low	50%	17%	33%

Low Risk			43%	14%	0%	71%	100%	100%	-	-	-
High Risk			0%	14%	100%	0%	0%	0%			
Unclear			57%	71%	0%	29%	0%	0%	-	-	-

NOTES: Blinding and Selective Reporting limited to global well-being using the FIQ; Other Bias limited to between-group baseline differences in global well-being using the FIQ.

### Participant Characteristics

A general description of the participants for each group from each study is provided in Table 2. The total number of participants was 473 (280 exercise, 193 control), ranging from 15 to 56 in the exercise groups ( ± SD, 40 ± 32) and 14 to 40 in the control groups ( ± SD, 28 ± 11). The percentage of dropouts ranged from 5.6% to 46.7% in the exercise groups ( ± SD, 27.9% ± 16.9%) and 0% to 63.0% in the control groups ( ± SD, 19.2% ± 22.0%). One study included six men (three exercise, three control) [9] while the remaining six studies were limited to women [8,10-14]. In total, approximately 99% of the participants were women. The within-study age of the participants ranged from 18 to 73 years in both the exercise and control groups while the mean between-group age ranged from 41 to 51 years in the exercise groups ( ± SD, 46.0 ± 3.7) and 42 to 52 years in the control groups ( ± SD, 48.0 ± 3.3). For race and ethnicity, one study reported that all participants were Caucasian [12] while another reported that greater than 90% of the participants were Caucasian with the remaining consisting of Aboriginals and Hispanics [13]. Another study appeared to consist entirely of Hispanics [14].

**Table 2:** General Characteristics of Studies.

Reference	N	Age (Years)	Gender (F/M)	Duration FM (Years)	Exercise Intervention
Da Costa et al. (2005)[8]	Ex: 39 Con (usual care): 40	Ex: 49.2 ± 8.7 Con: 52.3 ± 10.8	F	Ex:10.5 ± 8.4 Con: 11.2 ± 7.6	12 weeks home-based aerobic exercise, 60-120 min/week, 60-85% MHR; strengthening & stretching; compliance to aerobic exercise, 65.9%
Gowans et al.(2001)[9]	Ex: 27 Con (usual care): 23	Ex: 44.6 ± 8.7 Con: 49.8 ± 7.3	F (88%)/M	Ex: 9.6 ± 8.6 Con: 8.4 ± 7.6	23 weeks supervised, facility-based aerobic exercise, 3×/wk, 20 min/day, 60-75% MHR; compliance, 67%.
King et al. 2002)[10]	Ex: 46 Con (attention control): 39	Ex: 45.2 ± 9.4 Con: 47.3 ± 7.3	F	Ex: 7.8 ± 6.1 Con: 9.6 ± 7.9	12 weeks supervised, facility-based aerobic ex, 3×/wk, 10-40 min/day, 75%MHR
Kingsley et al. (2005)[11]	Ex: 15 Con (usual care): 14	Ex: 45 ± 9 Con: 47 ± 4	F	Ex: 9 ± 10 Con: 7 ± 5	12 weeks strength training, 11 ex, 2×/wk, 1 set, 8-12 reps, 40-80% 1RM
Munguia-Izquierdo & Legaz-Arrese (2007)[12]	Ex: 29 Con (usual care): 24	Ex: 50 ± 7 Con: 46 ± 8	F	Ex: 14 ± 10 Con: 14 ± 9	16 weeks supervised, facility-based ex, 3×/wk; strengthening (1-3 sets, 8-15 reps, 8-10 ex); aerobic (20-30 min, 50-80% MHR); compliance ≥ 75%
Schachter et al. (2003)[13]	Ex (sb): 56 Ex: (lb): 51 Con (monthly small group meetings to discuss fibromyalgia): 36	Ex (sb): 41.9 ± 8.6 Ex: (lb): 41.3 ± 8.7 Con: 42.5 ± 6.7	F	Ex (sb): 8.6 ± 6.0 Ex (lb): 8.8 ± 6.2 Con: 8.8 ± 5.0	16 weeks home-based, low-impact aerobic ex; short bout, 2×/day, 3×/wk, 5-15 min/session, 40-75% HRR; long bout, 1×/day, 3×/wk, 10-30 min/session, 40-75% HRR
Tomas-Carus et al. (2007) [14]	Ex: 17 Con (usual care): 17	Ex: 51 ± 10 Con: 51 ± 9	F	Ex: 24 ± 9 Con: 19 ± 8	12 weeks, supervised, facility-based aerobic and strengthening ex, 3×/wk; aerobic, 20 min/day, 65-75% MHR; compliance >95%

Notes: Description of groups and subjects from each study limited to those that met the inclusion criteria; N, number of subjects; age reported as mean () ± standard deviation (SD); F, females; M, males; Ex, Exercise; Con, Control; FM, fibromyalgia; MHR, maximum heart rate; 1RM, one-repetition maximum; HRR, heart rate reserve; lb, long bout; sb, short bout; min, minutes; wk, week; reps, repetitions.

In relation to medications, five studies reported that one or more participants were taking some type of medication(s) for fibromyalgia [8-11,14], although it is likely that all studies had one or more participants taking some type of medication(s).

The mean between-group duration of self-reported fibromyalgia symptoms ranged from 8 to 24 years in the exercise groups ( ± SD, 11.5 ± 5.4) and 7 to 19 years in the control groups ( ± SD, 11.1 ± 4.1) [8-14]. For the three studies and four groups in which information were available [8,9,13], the mean between-group duration since physician diagnosis of fibromyalgia ranged from 2.8 to 3.8 years in the exercise groups ( ± SD, 3.3 ± 0.5) and 3.6 to 4.9 years in the control groups ( ± SD, 4.2 ± 0.7).

One study reported that some participants smoked [13] while little information was provided regarding diet, including alcohol intake. In relation to exercise, six studies reported that none of the participants were exercising regularly prior to participating in the study [8,9,11-14] while one reported that some had been exercising prior to participation [10]. Five studies appeared to include one or more subjects who were overweight or obese [8,10-12,14], defined as a body mass index ≥ 25 kg/m^2^.

### Exercise Program Characteristics

A description of the characteristics of the exercise programs for each group from each study is shown in Table 2. Four groups from three studies focused on aerobic exercise [9,10,13], one study was limited to strengthening exercise [11], while three others included both aerobic and strengthening exercise [8,12,14]. For those studies in which data were available [9-14], frequency of training ranged from two to six sessions per week ( ± SD, 3 ± 1) with three times per week being the most common. The duration of within-group aerobic and/or strengthening exercise ranged from five to 50 minutes per session [9,10,12-14] while the mean between-group range was 12 to 24 minutes per session ( ± SD, 19.2 ± 5.1) [9,12-14]. Within-group intensity of aerobic training ranged from 40% to 85% of maximum heart rate (MHR) [8-10,12-14]. The one study that was limited to strengthening exercise reported a training intensity between 40% and 80% of one repetition maximum (1RM) [11]. Between-groups mean compliance, defined as the percentage of exercise sessions attended, ranged from 65.9% to more than 95.0% ( ± SD, 75.7% ± 13.5%) [8,9,12,14]. Four studies had participants perform exercise in a pool [9,10,12,14]. For those studies that reported data, four had participants perform supervised, facility-based exercise [9,10,12,14], another study with two exercise groups had participants perform unsupervised, home-based exercise [13] and another had participants perform primarily unsupervised, home-based exercise as well as four supervised sessions [9].

### Findings for Global Well-Being using the FIQ

#### Overall results

**Figure 2:**
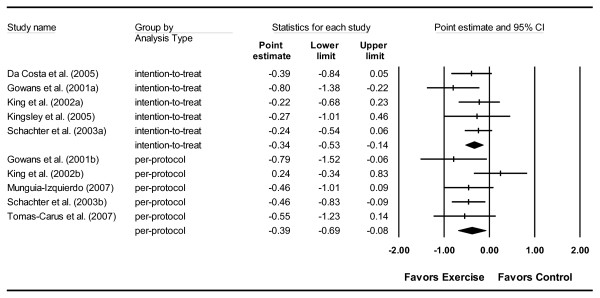
**Forest plot for changes in global-well being**. Forest plot for point estimate standardized effect size changes (Hedge's *g*) in global well-being derived from the total score on the FIQ and analyzed according to per-protocol analyses and intention-to-treat analyses. The black squares represent the standardized mean difference (Hedge's *g*) while the left and right extremes of the squares represent the corresponding 95% confidence intervals. The middle of each of the two black diamond's represents the overall standardized mean difference (Hedge's *g*) for each type of analysis (per-protocol and intention-to-treat) while the left and right extremes of the diamonds represent the corresponding 95% confidence intervals.

A small, statistically significant improvement in global well-being was observed for both per-protocol and intention-to-treat outcomes in the exercise groups (Figure 2). No statistically significant within-group heterogeneity was found (per-protocol, Q_w _= 6.04, *p *= 0.20, *I*^2 ^= 33.8%; intention-to-treat, Q_w _= 3.19, *p *= 0.53, *I*^2 ^= 0%) and no statistically significant differences between per-protocol and intention-to-treat outcomes were observed (Q_b _= 0.07, *p *= 0.80). No study was excluded from the meta-analysis because of a lack of data (selective reporting) for the FIQ.

Using the CLES, 61% (per-protocol analysis) and 60% (intention-to-treat analysis) of participants in the exercise group would score higher than control group participants if chosen at random. Changes were equivalent to an odds ratio (OR) improvement of 51% (OR = 0.49, 95% CI, 0.27 to 0.87) based on the per-protocol approach and 47% (OR = 0.53, 95% CI, 0.36 to 0.77) based on intention-to-treat analysis. Relative to baseline values for the FIQ, exercise minus control group improvements were equivalent to 8.2% for per-protocol analysis and 7.2% for intention-to-treat analysis.

#### Publication bias

When per-protocol results were adjusted for potential publication bias (one imputation), results remained statistically significant (*g*, -0.32, 95% CI -0.62 to -0.02). No adjustment for publication bias was needed for intention-to-treat results.

#### Sensitivity analysis

**Figure 3:**
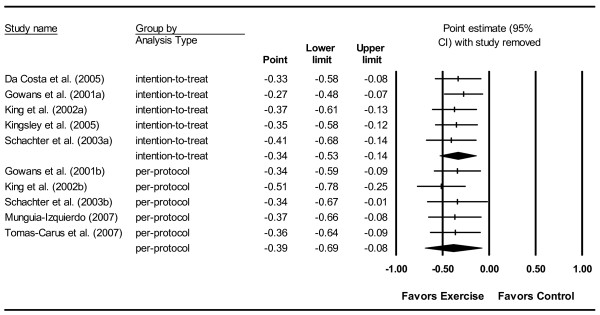
**Forest plot for changes in global-well being with each study deleted once**. Point estimate standardized effect size changes (Hedge's *g*) in global well-being derived from the total score on the FIQ and analyzed according to per-protocol and intention-to-treat and analyses with each study deleted from the model once. The black squares represent the standardized mean difference (Hedge's *g*) while the left and right extremes of the squares represent the corresponding 95% confidence intervals. The middle of each of the two black diamond's represents the overall standardized mean difference (Hedge's *g*) for each type of analysis (per-protocol and intention-to-treat) while the left and right extremes of the diamonds represent the corresponding 95% confidence intervals.

With each outcome deleted from the model once, results remained statistically significant across all deletions for both per-protocol and intention-to-treat analyses (Figure 3).

#### Cumulative meta-analysis

**Figure 4:**
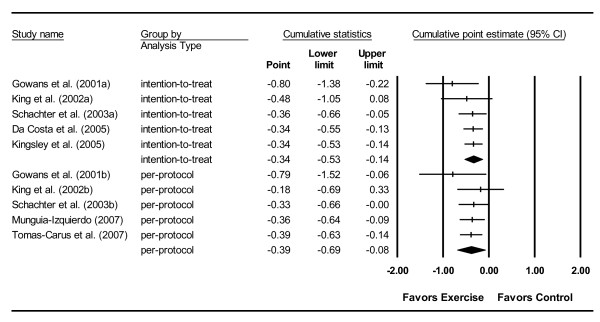
**Cumulative meta-analysis for changes in global well-being**. Cumulative meta-analysis ranked by year, for point estimate standardized effect size changes (Hedge's g) in global well-being derived from the total score on the FIQ and analyzed according to per-protocol and intention-to-treat and analyses. The black squares represent the standardized mean difference (Hedge's g) while the left and right extremes of the squares represent the corresponding 95% confidence intervals. The middle of each of the two black diamond's represents the overall standardized mean difference (Hedge's g) for each type of analysis (per-protocol and intention-to-treat) while the left and right extremes of the diamonds represent the corresponding 95% confidence intervals. Studies are added one at a time according to date of publication and the results summarized as each new study is added.

When ranked by year, cumulative meta-analysis demonstrated that results have been statistically significant since 2003 for both per-protocol and intention-to-treat analyses (Figure 4).

## Discussion

Using intention-to-treat and per-protocol analyses, the results of this study suggest that exercise, as assessed by the FIQ, improves global well-being in community-dwelling women with fibromyalgia. These findings are reinforced by the fact that results have been consistent since 2003 and that all findings remained statistically significant when each study was deleted from the model once. The fact that no statistically significant difference was found between per-protocol and intention-to-treat outcomes is important given that recent research has suggested that excluding participants from the analysis in randomized controlled trials often results in biased estimates of treatment effects when the results of these studies are pooled in systematic reviews [21]. These overall findings are also similar to an earlier meta-analysis that used multiple measures to assess global well-being and did not differentiate between per-protocol and intention-to-treat analyses [7]. The similar findings between both reviews are important since it is not uncommon for the results of systematic reviews on the same topic to differ [43]. This should give practitioners more confidence in the expected benefits of exercise on global well-being in women with fibromyalgia.

The results of this study suggest that a low risk of bias exists for incomplete outcome data, selective reporting, and between-group differences in baseline measures for the FIQ. In contrast, a high risk of bias was observed for blinding while sequence generation and allocation concealment was unclear in more than 50% of the studies. Consequently, it would seem appropriate to suggest that future exercise intervention studies apply appropriate blinding procedures as well as applying and clearly reporting adequate methods for sequence generation and allocation concealment. However, these results should be interpreted cautiously. In order to provide a greater degree of flexibility across a variety of different research domains, a substantial degree of subjectivity is allowed in the use of the Cochrane Collaborations bias assessment tool [32]. Consequently, other individuals assessing the same studies may arrive at different conclusions regarding the risk of bias across the different domains. For example, while studies were considered high risk if they did not blind participants, personnel and outcome assessors to group assignment, others may have chosen to classify studies as low risk if only the outcome assessor or outcome assessor and other study personnel were blinded. This approach would appear plausible given that it is extremely difficult, if not impossible, to blind participants to group assignment in exercise intervention studies. Nevertheless, the risk for bias still exists and may be especially problematic for subjective measures such as the FIQ as opposed to a more objective measure such as body weight. Thus, this form of bias is not a study-specific problem, but rather, inherent to exercise intervention studies. In addition, while a low risk of bias was found for selective reporting of outcomes, only one outcome was the primary focus of the current meta-analysis. In contrast, the inclusion of multiple outcomes, for example changes in lipids and lipoproteins, might result in more studies being classified as high versus low risk. The same may be true for the domain "other forms of bias" as the present investigation limited this domain to between-group differences in baseline values in global well-being as assessed by the FIQ. Finally, while selective reporting should ideally be determined by examining the study protocol [32], the identification of study protocols was not possible in the current investigation. Therefore, the recent recommendations of Hartling et al. [33] were adhered to whereby the degree of risk was based on whether the FIQ was described in the methods section and reported in the results. By adhering to the study protocol approach, all studies would have been categorized as having an unclear versus low risk for bias.

It has recently been suggested that a 14% change in the total score on the FIQ is clinically relevant [44]. While the relative changes in the current meta-analysis ranged between 7.2% and 8.2%, they may still be clinically important given that no gold standard exists for determining such. In addition, numerous other benefits can be derived from exercise while the risks associated with participation in a program of regular exercise are minimal [45]. Given the former, it would seem plausible to suggest that participation in exercise programs similar to those included in the current meta-analysis should yield the same improvements in global well-being. However, the ability to statistically examine the issue of dose-response, including intensity, was not possible because of the small number of studies included. As suggested by the recent Department of Health and Human Services Physical Activity Guidelines Advisory Committee Report [25], additional studies are needed to determine the optimal length, frequency, intensity and duration necessary for maximizing benefits among adults with arthritis and other rheumatic conditions, including fibromyalgia. This includes an examination of how to progressively increase the dose of activity, including intensity, so that maximum benefits can be obtained. Until such information is available, it may be prudent to follow the general guidelines of the American College of Sports Medicine regarding exercise [23,24].

While the results of this meta-analysis are encouraging, they must be interpreted while taking other issues into account. For example, it is probably not possible to generalize the findings of this meta-analysis to men with fibromyalgia given that few studies included men. Despite the lower prevalence in men [2], it would seem appropriate to suggest that future research on the effects of exercise on global well-being in men is necessary.

Because of a lack of statistically significant heterogeneity (p > 0.10) and inconsistency (<50%) the results of studies in which the content of the exercise intervention varied, i.e., aerobic and/or strengthening exercise, supervised and/or non-supervised sessions, home and/or facility based exercise, were pooled. While a lack of heterogeneity and inconsistency does not exclude one from conducting sensitivity and/or subgroup analyses, this was not possible given the small number of studies included as well as the lack of data available for the variables of interest. Given the former, it is suggested that future randomized controlled trials include and compare exercise interventions of varying content so as to determine their potential impact on global well-being in adults with fibromyalgia. In addition, future studies should report complete data for these variables as they may have an effect on global well-being.

To reduce potential bias, the current meta-analysis was limited to only those studies in which the FIQ was used. However, it is possible that such a limitation could have caused selection bias. One possible alternative would have been to include studies that used other measures for assessing global well-being and then perform some type of sensitivity or subgroup analyses to see what effect, if any, the inclusion of such may have had on global well-being outcomes. However, it's important to realize that these types of analyses are observational in nature because studies are not randomly assigned to moderators [46]. Consequently, such analyses do not support causal inferences. In meta-analysis, causal inferences can only be inferred from the overall results of randomized controlled trials [47].

Finally, the exact search strategy for the identification of randomized trials as recommended by the Cochrane Collaboration was not followed [32]. Consequently, the possibility exists that relevant studies may have been missed. However, this seems unlikely given the exhaustive search methods employed.

## Conclusions

The results of this study suggest that exercise improves global well-being in community-dwelling women with fibromyalgia. However, additional research on this topic is needed, including research in men as well as optimal exercise programs for improving global well-being in adults.

## Competing interests

The authors declare that they have no competing interests.

## Authors' contributions

GAK was responsible for the conception and design, acquisition of data, analysis and interpretation of data, drafting the initial manuscript and revising it critically for important intellectual content. KSK was responsible for the conception and design, acquisition of data, and reviewing all drafts of the manuscript. JMH was responsible for the conception and design, interpretation of data and reviewing all drafts of the manuscript. DLJ was responsible for the conception and design, interpretation of data and reviewing all drafts of the manuscript. All authors read and approved the final manuscript

## Pre-publication history

The pre-publication history for this paper can be accessed here:

http://www.biomedcentral.com/1471-2458/10/198/prepub

## Supplementary Material

Additional file 1 **Supplement 1, User Queries for Original Database Searches**. This supplementary material contains the user queries used for our electronic database searches Click here for file
