# Supplementary material for: Exercise and global well-being in community-dwelling adults with fibromyalgia: a systematic review with meta-analysis
Source: BMC Public Health. 2010 Apr 20;10:198. doi: 10.1186/1471-2458-10-198 (PMC2874776; doi:10.1186/1471-2458-10-198)
Supplement: Additional file 1 — Supplement 1, User Queries for Original Database Searches. This supplementary material contains the user queries used for our electronic database searches [file 1471-2458-10-198-S1.DOC]

**Supplement 1**

**User Queries for Original Database Searches**

**PubMed**

exercise AND arthritis OR exercise AND osteoarthritis OR exercise AND fibromyalgia OR exercise AND rheumatoid arthritis OR exercise AND lupus OR exercise AND ankylosing spondylitis OR exercise AND gout OR exercise AND rheumatic AND ((“1980/01/01”[PDat]:“2008/01/01”[PDat]) AND (Humans[Mesh]) AND (Randomized Trial [ptyp]) AND adult[MeSH]))

**EMBASE**

‘exercise’/exp AND ‘arthritis’/exp OR ‘exercise’/exp AND ‘osteoarthritis’/exp OR ‘exercise’/exp AND ‘fibromyalgia’/exp OR ‘exercise’/exp AND rheumatoid AND ‘arthritis’/exp OR ‘exercise’/exp AND ‘lupus’/exp OR ‘exercise’/esp AND ankylosing AND ‘spondylitis’/exp OR ‘exercise’/exp AND ‘gout’/exp OR ‘exercise’/exp AND rheumatic AND [randomized controlled trial]/lim AND [humans]/lim AND ([adult]/lim OR [aged]/lim) AND [embase/lim AND [01-01-1980]sd NOT [01-01-2008]sd AND [1980-2008]py

**Cochrane Central Register of Controlled Clinical Trials**

“exercise AND arthritis OR exercise AND osteoarthritis OR exercise AND fibromyalgia OR exercise AND rheumatoid arthritis OR exercise AND lupus OR exercise AND ankylosing spondylitis OR exercise AND gout OR exercise AND rheumatic in Title, Abstract or Keywords, from 1980 to 2007 in The Cochrane Central Register of Controlled Trials”

**CINAHL**

Search Terms – TI (exercise AND arthritis OR exercise AND osteoarthritis OR exercise AND fibromyalgia OR exercise AND rheumatoid arthritis OR exercise AND lupus OR exercise AND ankylosing spondylitis OR exercise AND gout OR exercise AND rheumatic) or AB (exercise AND arthritis OR exercise AND osteoarthritis OR exercise AND fibromyalgia OR exercise AND rheumatoid arthritis OR exercise AND lupus OR exercise AND ankylosing spondylitis OR exercise AND gout OR exercise AND rheumatic)

Limiters and Expanders – Publication year from: 1980-2008; Published Date from 198001-200801; Publication Type: Clinical Trial, Journal Article; Age Groups: All Adult

**SPORTDiscus**

Search Terms – TI (exercise AND arthritis OR exercise AND osteoarthritis OR exercise AND fibromyalgia OR exercise AND rheumatoid arthritis OR exercise AND lupus OR exercise AND ankylosing spondylitis OR exercise AND gout OR exercise AND rheumatic) or AB (exercise AND arthritis OR exercise AND osteoarthritis OR exercise AND fibromyalgia OR exercise AND rheumatoid arthritis OR exercise AND lupus OR exercise AND ankylosing spondylitis OR exercise AND gout OR exercise AND rheumatic)

Limiters and Expanders – Year published from: 1980-2008; English Abstract Available; Publication Type: Journal Article

**Dissertation Abstracts Online**

(ti: exercise AND ti: arthritis OR ti: exercise AND ti: osteoarthritis OR ti: exercise AND ti: fibromyalgia OR ti: exercise AND (ti: rheumatoid and ti: arthritis) OR ti: exercise AND ti: lupus OR ti: exercise AND (ti: ankylosing and ti: spondylitis) OR ti: exercise AND ti: gout OR ti: exercise AND ti: rheumatic) or (ab: exercise AND ab: arthritis OR ab: exercise AND ab: osteoarthritis OR ab: exercise AND ab: fibromyalgia OR ab: exercise AND (ab: rheumatoid and ab: arthritis) OR ab: exercise AND ab: lupus OR ab: exercise AND (ab: ankylosing and ab: spondylitis) OR ab: exercise AND ab: gout OR ab: exercise AND ab: rheumatic) and yr: 1980-2008
